# Annual Opening

**Published:** 1887-11

**Authors:** 


### ANNUAL OPENING.

The medical colleges of this city have begun their annual course of instruction this year under favorable auspices.

The throng of students in attendance upon the lectures is undiminished—indeed, shows marked increase in numbers and in the amount of preparatory training and preparation.

In view of the crowded condition of the ranks of the medical profession all over the South and West, the large attendance at the various colleges is somewhat surprising, and demonstrates that medicine as a pursuit still maintains its popularity and retains a strong hold upon public esteem and confidence.

Enlightened public opinion in all ages has assigned to the skilled and honorable physician an elevated place in the social scale. It is not wonderful that large numbers of ambitious young men of education should aspire to the privilege of fighting the battle of life upon so high a plane.

The sprightly newspaper reporters, who every year, after the college commencements, startle the public by the announcement of "hundreds of young fledgelings licensed to kill," are little aware of the years of patient study, of wearing, anxious toil the young licentiate has had to expend to qualify him for the tremendous responsibility. The reporter's ideas of medical attainments are probably derived from the comedies of Molliere, or the humorous squibs of satirists, for whom physicians have always been a favorite target. His ideal of the medical student and of student life has been formed upon the model of Bob Sawyer and Ben Allen, all redolent with the fumes of tobacco-smoke and the beer-keg.

He is not supposed to know that the widening circle of medical science now demands of those who practice it as an art an extent, a variety and a minute accuracy of information upon a multiplicity of subjects not dreamed of in his philosophy.

The medical students in our colleges now cannot afford to be night-roysterers or day-idlers. The number and complexity of the subjects pertaining to his course and daily pressed upon his attention demand every moment of his time.

The final examinations of the students, in accordance with the reasonable expectation of the public, is becoming every year more rigorous, and to pass that ordeal exacts such continuous and earnest effort during the whole period of pupilage as to leave no room for dissipation. In this city, at least, medical students are characterized by quiet, modest, gentlemanly demeanor and diligence in their studies. It is believed that the present class will "beat the record."

